# Emerging role of Protein Kinase CK2 in Tumor immunity

**DOI:** 10.3389/fonc.2022.1065027

**Published:** 2022-12-01

**Authors:** Leichong Chen, Sijia Zhang, Qianwen Li, Junyu Li, Huilin Deng, Sheng Zhang, Rui Meng

**Affiliations:** ^1^ Cancer Center, Union Hospital, Tongji Medical College, Huazhong University of Science and Technology, Wuhan, China; ^2^ Department of Radiation Oncology, Jiangxi Cancer Hospital, Nanchang, Jiangxi, China

**Keywords:** protein kinase CK2, tumor immunity and immunotherapy, tumor microenvirenment, PD-L1, signal pathway

## Abstract

Protein kinase CK2, a conserved serine/threonine-protein kinase, is ubiquitous in cells and regulates various intracellular processes, especially in tumor cells. As one of the earliest discovered protein kinases in humans, CK2 plays a crucial role in phosphorylating or associating with hundreds of substrates to modulate several signaling pathways. Excellent reviews have reported that the overexpression of CK2 could be observed in many cancers and was closely associated with tumor occurrence and development. The elevation of CK2 is also an indicator of a poor prognosis. Recently, increasing attention has been paid to the relationship between CK2 and tumor immunity. However, there is no comprehensive description of how CK2 regulates the immune cells in the tumor microenvironment (TME). Also, the underlying mechanisms are still not very clear. In this review, we systematically summarized the correlation between CK2 and tumor immunity, primarily the effects on various immune cells, both in innate and adaptive immunity in the TME. With the comprehensive development of immunotherapy and the mounting transformation research of CK2 inhibitors from the bench to the clinic, this review will provide vital information to find new treatment options for enhancing the efficacy of immunotherapy.

## 1 Introduction

### 1.1 CK2 structure, functions, and relevance to cancer development

Protein kinase CK2, formerly known as casein kinase II, is a constitutively active Ser/Thr protein kinase that exists as a tetramer in cells. It contains two catalytic subunits (α or α’) and two regulatory subunits (β) which lead to three different conformations: α2β2,α’2β2, αα’β2 (1). Among these subunits, the two catalytic subunits are encoded by *CSNK2A1* and *CSNK2A2*, respectively. The regulatory subunit is encoded by *CSNK2B*, which regulates the selection of the substrates and enhances the catalytic subunit stability (2, 3).

CK2 is commonly expressed in all eukaryotic cells and phosphorylates hundreds of substrates to modulate the physiological activities in various cells, including tumor cells (4–6). Aberrant CK2 activity is related to tumor-promoting responses, which leads to the activation of various oncogenic signaling pathways. Also, it can cause multiple typical landmarks of cancers, including cell proliferation, angiogenesis, invasion, and metastasis (7–9). CK2 also participates in DNA damage and repair, the ER-stress response, altering cell morphology and promoting cellular transformation (10–13). Studies have subsequently revealed that the elevation of CK2 expression has been observed in most cancers, such as lung, glioblastoma, breast, ovarian, and melanoma, as well as in blood cancers (14). And the overexpression of CK2 correlates with tumor invasion and acts as a poor prognosis indicator (15). These information emphasizes that targeting CK2 is currently receiving more attention as a therapeutic approach for tumors. CX-4945 (a CK2 inhibitor) is currently approved by FDA(Food and Drug Administration) for the treatment of cholangiocarcinoma. And beneficial effects of various CK2 inhibitors have also been observed *in vitro* experiments, many of them are currently in clinical trials for solid cancers (16–18).

## 2 The relationship between CK2 and immune response in the tumor microenvironment (TME)

Evidence shows that CK2 has a critical role in innate and adaptive immune cells in various inflammatory diseases such as glomerulonephritis (19), autoimmune encephalomyelitis (EAE) (20) and allergic contact dermatitis (21). E.N. Benveniste et al. have also conducted numerous studies about the significant role of CK2 in regulating various immune cells in a variety of inflammatory diseases (22–24). These excellent findings provide essential evidence for the importance of CK2 in the pathogenesis of inflammatory responses.

Based on the increasing evidence highlighting the function of CK2 in promoting inflammatory diseases, we suggest that CK2 could also have unexpected effects on tumor immunity. Growing evidence has shown that CK2 also has a practical impact on diverse immune cells in the tumor microenvironment (TME).

Protein kinase CK2 has been found to be well associated with several critical oncogenic signaling pathways modulating the development of immune cells in the tumor microenvironment(TME) (22, 25). For instance, CK2 activity improved the signals, including NF-κB, JAK/STAT, COX-2, HIF-1α, ERK, AKT, and Wnt, and suppressed the Notch and Ikaros pathways (26–32). Given that these signaling pathways are closely associated with tumor immunity.CK2 may potentially affect these immune cells’ growth and development by regulating these critical signaling pathways in TME. Some of the perspectives have already been demonstrated.

### 2.1 The role of CK2 in the innate immune system

#### 2.1.1 Myeloid-derived suppressor cells (MDSCs) and tumor-associated macrophages (TAMs).

Several reports have shown that various oncogenic signaling pathways modulated the development of immune cells in the TME. The Notch signal pathway is a critical tumor suppressor and controls diverse cells fate decisions, including immune cells growth, differentiation, and cell cycle progression (33, 34), especially in myeloid cells, such as myeloid-derived suppressor cells (MDSC) and dendritic cells (DCs) in the tumor microenvironment (35, 36). Once the cognate ligand combines to the Notch receptor, the Notch signal will be activated after a sequence of events. The Notch receptor is cleaved by ADAM metalloproteases and γ-secretase complex at specific sites, respectively, and releases the Notch intracellular domain (NICD). The NICD will translocate to the nucleus to bind with CSL(the transcriptional repressor CBF1) and induce the transcription of Notch (37, 38).

**Figure 1:**
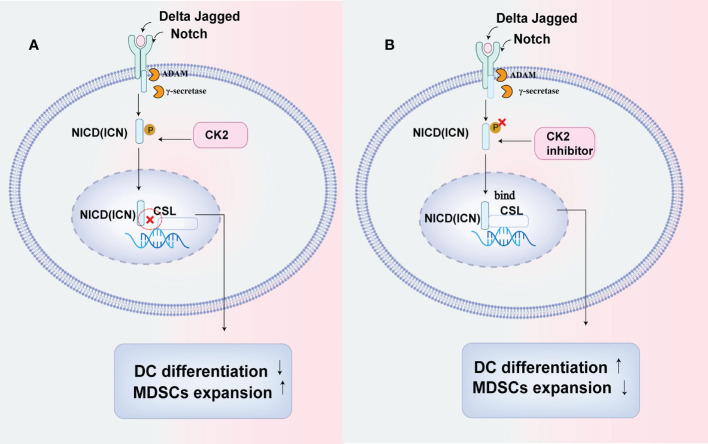
CK2 can affect the differentiation of myeloid cells by down-regulating the Notch signal pathway. **(A)** CK2 can phosphorylate the Notch receptor NICD (Notch intracellular domain)and decrease the binding of NICD to transcriptional repressor CSL, resulting in reduced notch signal transcriptional activity, which will lead to the defective DC cells differentiation while increasing the accumulation of immunosuppressive cells including PMN-MDSC and tumor-associated macrophages (TAMs) in tumors. **(B)** CK2 inhibitor can restore the activation of Notch signal in myeloid cells and promote NICD to combine with CSL to enhance DC cells differentiation while reducing the production of PMN-MDSCs and tumor-associated macrophages (TAMs) by blocking their differentiation.

Several CK2 phosphorylation sites of the Notch signal have been illustrated by Ranganathan et al. (39). Cheng et al. reported that CK2 downregulated the Notch signal pathway in myeloid cells in several tumors. They used three cell lines: EL4 (lymphoma), CT26 (colon cancer), and MethA (sarcoma) and illustrated that CK2 activity phosphorylated the Notch receptor NICD(Notch intracellular domain)and decreased the binding of NICD to the transcriptional repressor CSL, which led to a reduced Notch signal transcriptional activity. And the downregulation of Notch resulted in defective DC cells differentiation and a promotion in the accumulation of immunosuppressive cells, including PMN-MDSC and tumor-associated macrophages (TAMs) in tumors (35, 40). While a selective CK2 inhibitor, tetrabromocinnamic acid(TBCA)restored the Notch signal pathway in myeloid cells to enhance DC cells differentiation and block the differentiation of PMN-MDSC (35) (**Figure 1**).

As we all know, the accumulation of immature myeloid cells, such as MDSCs, promotes the immunosuppression in TME. And DCs will lose their ability to prime CD8+ T cells in an immature state. Therefore, CK2 activity has tumor-promoting properties in TME by regulating the Notch signal in myeloid cells (41), which suggests that targeting CK2 can be beneficial in improving anti-tumor immunity to treat cancers.

Also, evidence of the crosstalk between CK2 and macrophages has also been illustrated by Ayumi et al. They demonstrated that CK2 inhibitors (BMS-211 and BMS-595) reduced the population of PMN-MDSC in the spleen and TAMs in the LLC (lung carcinoma) TB mice. To investigate the specific mechanism, they isolated these cells from TB mouse spleen and found that CK2 inhibitors can block the differentiation of the precursors of granulocytes and macrophages and dramatically suppress the proportion of macrophages generated from monocytic cells (40).

#### 2.1.2 Dendritic cells (DCs)

The crosstalk between CK2 and dendritic cells (DCs) has also been illustrated recently. Marisa et al. demonstrated that the RNA Pol III activation was controlled by CK2, which is required for DCs to prime T cell (42). In addition, apart from tumor cells, PD-L1 is also expressed in dendritic cells (DCs) and macrophages, DCs may be a vital target for PD-L1 antibodies (43, 44). Zhao et al. demonstrated that CK2 activity played a key role in targeting PD-L1 expression in DC cells. And CK2 inhibitor (CX-4945) downregulated the expression of PD-L1 on tumor-associated DCs and activated DC cells’ function to prime T cells (45). These findings suggest that CK2 inhibitors may exert its anti-tumor effect in various aspects.

#### 2.1.3 NK cells

NK cells can secrete perforin, granzyme, and other cytotoxic lytic particles as well as express TNF superfamily members, such as FAS Ligand (FASL) and TNF-related apoptosis-inducing Ligand (TRAIL), to induce the apoptosis of target cells (46).

It is reported that the inhibition of CK2 can augment NK cells antitumor function by enhancing their cytotoxicity. Kim et al. investigated the effects of TBB (a CK2 inhibitor) on NK cells in three cancer cell lines, HepG2, Hep3B(Hepatocellular carcinoma), and HeLa. They showed that the inhibition of CK2 can increase NK cell-mediated cancer-killing through a particle-dependent process (46, 47). Like NK cells, cytotoxic T cells (CTL) can use the same way to kill tumor cells. We can therefore speculate that CK2 inhibitors may also have a vital function in cytotoxic T cells to improve their anti-tumor ability.

### 2.2 The Role of CK2 in the adaptive immune system

#### 2.2.1 CD4^+^ T cells, CD8^+^T cells, Treg cells

Reports of the crosstalk between CK2 and adaptive immune cells have been provided by Nelson N et al. CK2 can affect the development of CD4^+^ T cells, CD8^+^ T cells and Treg cells by regulating the Ikaros pathway (48). Ikaros is expressed in various hematopoietic stem cells, lymphoid, and some myeloid cells. It is essential for the normal development of lymphocytes and other blood cell lineages (49–51). Evidence shows that CK2 can phosphorylate the Ikaros signal at multiple sites (52, 53). And the phosphorylation of the Ikaros signal leads to its ubiquitin-mediated proteasomal degradation and induces the downregulation of Ikaros activity and expression (48, 54, 55). In contrast, protein phosphatase 1 (PP1) can dephosphorylate the Ikaros pathway to maintain its stability and activity (52). Therefore, the harmony of CK2 and PPI plays an essential role in the state of the Ikaros signal.

**Figure 2:**
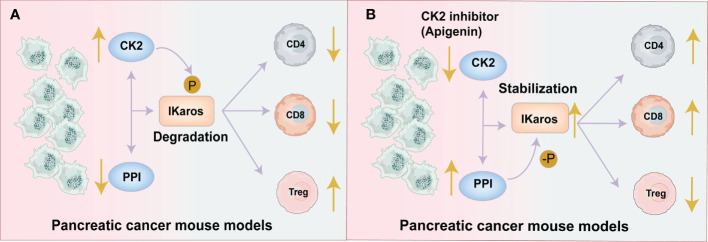
CK2 activity decreases the effector CD4^+^and CD8^+^T cells and increases Tregs by regulating the Ikaros signal. **(A)** Pancreatic Tumor Microenvironment without Apigenin (API): CK2 activity phosphorylates the Ikaros signal and promotes its degradation, leading to the downregulation of Ikaros expression. The reduction in Ikaros expression causes an increase in Tregs and a decrease of effector CD8^+^T and CD4^+^ T cells. This leads to a decreased antitumor effect. **(B)** Pancreatic Tumor Microenvironment with API: API targets and reduces the activity of CK2 and restores the expression of Ikaros, increasing the effector CD8^+^T and CD4^+^ cells while reducing Tregs to promote antitumor immunity.

Nelson N et al. showed that in pancreatic cancer mouse models, CK2 activity phosphorylates the Ikaros signal and leads to an increase in Tregs while a reduction in effector CD8^+^T and CD4^+^ T cells in TME. While Apigenin (API: a CK2 inhibitor) targets and reduces the activity of CK2 to restore the expression of Ikaros and increases the effector CD8^+^T and CD4^+^ cells while reduces the production of Tregs to promote the antitumor immunity (48, 56). (**Figure 2**). Also, Apigenin increases the function of CD8+ T cells to release IFN-γ and promotes better activation of allogeneic CD8+ T cell responses in spleen cells (48, 56). Therefore, CK2 inhibitors may be a potential therapeutic agent for stabilizing the Ikaros signal and maintaining T cell homeostasis to enhance antitumor immunity.

In addition, E.N. Benveniste et al. also demonstrated the critical role of CK2α in regulating the activation, proliferation, differentiation and function of normal CD8^+^T cells. CK2 is required for the glycolysis and oxidative respiration of CD8^+^T cells (57). However, the role of CK2 in the cytotoxic effect of CD8 ^+^T cells in tumor microenvironment has still not very clear.

#### 2.2.2 B cells

The function of protein kinase CK2 in the development and differentiation of normal B cells has also been illustrated by E.N. Benveniste et al. They found that CK2α deficiency in B cells causes the abnormal accumulation of MZB cells (marginal zone B cells), which was associated with the reduction of BCR signaling and increased Notch2 signaling (58). While the CK2 activation promotes the differentiation of MZB cells into plasma cells (58).

In addition, CK2 is significantly upregulated in a variety of hematological neoplasms, particularly in multiple myeloma(MM) (59). Francesco et al. illustrate that CK2 regulates the transcriptional activity of NF-κB signal by degrading its IκBα subunits in multiple myeloma cells. Also, TBB (a CK2 inhibitor) attenuates the activation of the IL-6-dependent STAT3 pathway in MM cells (60). Similarly, Zhao et al. reported that apigenin (a CK2 inhibitor) suppressed the transcriptional activity of several signal pathways, such as AKT, ERK, STAT3 and NF-κB and downregulated the expression of several anti-apoptotic proteins, to induce tumor cells apoptosis in multiple myeloma cells (61).

Multiple myeloma (MM) is characterized by the accumulation of malignant plasma cells in the bone marrow. Studies have shown that CK2 can regulate the proliferation and differentiation of normal B cells and promote the differentiation of MZB cells (marginal zone B cells) into plasma cells (58). Here, we speculate that CK2 inhibitor/knockout CK2 may also have a potential role in inhibiting the differentiation of MZB cells into plasma cells to suppress the malignant proliferation of MM cells. That is worthy of further study and may provide novel ideas for treating multiple myeloma.

## 3 The relationship between CK2 and the cytokines released by tumor cells or immune cells in TME

Inflammation plays a vital role in carcinogenesis. Evidence has shown that a variety of tumors can be caused by long-term chronic inflammation. And immune cells can release cytokines to induce the inflammatory microenvironment which can produce carcinogenesis (62). Apart from immune cells, tumor cells also secrete various cytokines such as TNF、GM-CSF、IL-6、IFN-γand MCP-1. These tumor-derived factors(TDF) establish an inflammatory microenvironment and modulate multiple immune cells in the TME, especially the expansion and recruitment of MDSC into the TME (63–65). And it will predispose to tumorigenesis and promote tumor progression (63, 66–68).

Krystal et al. illustrated that in mouse pancreatic tumor cells, apigenin(API: a CK2 inhibitor) could suppress the production of tumor-derived factors (TDF), including GM-CSF、IL-6、IFN-γ and MCP-1 to modulate immune cells development and promote tumor regression through enhancing the SHIP-1 expression (69). SH2-containing inositol phosphatase-1(SHIP-1) is mainly expressed in hematopoietic cells. And it is reported that SHIP-1 can also regulate immune cells differentiation, proliferation and migration by modulating many signaling pathways, especially for MDSCs, macrophages, DCs, and T cells (69–72).

**Figure 3:**
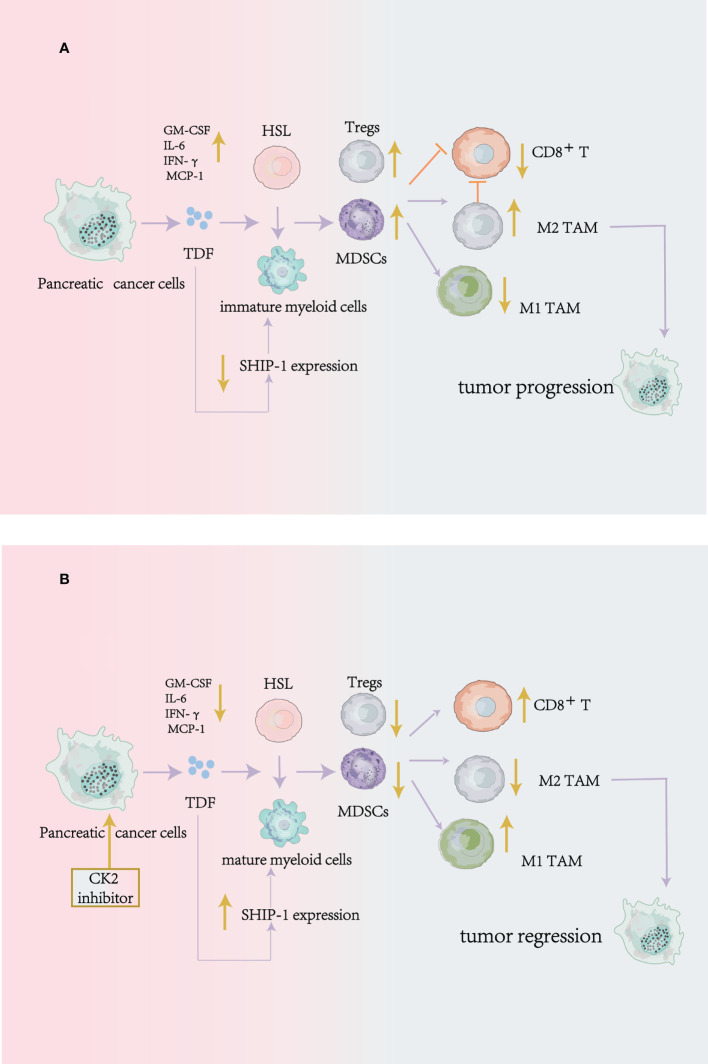
CK2 can regulate the production of tumor-derived cytokines to modulate antitumor immune responses in mouse pancreatic cancer cells. **(A)** without Apigenin (API: a CK2 inhibitor): Pancreatic cancer cells release tumor-derived factors (TDF), leading to a reduction of SHIP-1 expression, which causes hematopoietic stem cells (HSC) to become immature myeloid cells. The result is that the expansion of MDSCs, Tregs, and M2 phenotype TAM (pro-tumor) in the TME, on the contrary, the reduction of effector CD8 +T cells to inhibit antitumor immunity. **(B)** with Apigenin: apigenin can suppress the production of tumor-derived factors (TDF) produced by pancreatic cancer cells and enhance the SHIP-1 expression to reduce MDSCs and Tregs expansion, induce TAM to an M1 phenotype(antitumor) and increase the function of CD8+T cells in the TME to enhance antitumor immunity.

Krystal et al. demonstrated that the significant increase of SHIP-1 expression could be found in mouse spleen cells after CK2 inhibitor treatment. And the CK2 inhibitor suppressed the amplification of MDSCs and Tregs and enhanced the proportion of M1-like TAM (anti-tumor effect). Also, it promoted the percentage of CD8^+^ T cells and its function to release IFN-γ in the TME to induce antitumor immunity and tumor regression (69) (**Figure 3**).

Other reports of the crosstalk between CK2 and the cytokines in the tumor microenvironment have also been provided by Hagen Kulbe et al. It was reported that CK2 regulated the release of inflammatory cytokines to modulate the immune cells and the angiogenesis in ovarian cancer (73). They demonstrated that CK2 was associated with the TNF network, a network consisting of TNF-α and other cytokines which was released by ovarian cancer cells. The TNF network acts in a paracrine manner in the tumor microenvironment to affect the angiogenesis and immune cells infiltration (74, 75). Reports show that the CK2α knockdown or inhibition of CK2 (CX-4945) can significantly reduce the release of TNF, IL-6 and VEGF cytokines in ovarian cancer cells by suppressing the JAK/STAT3 and NOTCH signal pathway (73). And the levels of these cytokines are the key factors to affect tumor angiogenesis and immune cells infiltration in the TME (67, 76). In addition, reports have shown that the expression of IL-6 can be regulated by CK2 in inflammatory breast cancer (77), and IL-6 is an indicator of a poor prognosis (78). Also, recent findings have shown that IL-6 blockade promotes anti-CTLA-4 therapeutic role in the colon carcinoma model (79).

From the above, we can conclude that CK2 also has a critical effect on regulating the production of cytokines to modulate the function of immune cells in the tumor microenvironment(TME). Therefore targeting CK2 is a promising way to enhance antitumor immunity.

## 4 The relationship between CK2 and immune checkpoint receptor

The emergence of immune checkpoint inhibitors has significantly changed the cancer treatment paradigm and has achieved significant therapeutic effects in a variety of tumors. However, many patients still fail to respond to immunotherapy. A large number of literatures have reported that PD-L1 modulation is vital to improve the clinical response to anti–PD-1/PD-L1 treatment. Reports show that the inhibition of EGFR and/or CK2α can significantly reduce the expression of PD-L1 (80). Zhao et al. demonstrated that CK2 phosphorylated PD-L1 and prevented its ubiquitinated degradation to stabilize it on tumor cell. In contrast, CK2 inhibitors (CX-4945) can promote the degradation of PD-L1 reduce its level on tumor cells (45). They did a pan-cancer research, including lung cancer, breast cancer, prostate cancer, lymphoma, and so on. Apart from tumor cells, CK2 also regulates PD-L1 expression on dendritic cells. We know that CD80 on DC cells can interact with CD28 on T cells to initiate T cell activity. CK2 inhibitor can downregulate PD-L1 expression and enhance the level of CD80 on tumor-associated DCs and promote the CD80/CD28 interaction to enhance T-cell priming to inhibit tumor progression (45). Also, the combination treatment of CK2 inhibitors and immune checkpoint inhibitors can synergistically impede tumor growth, such as CTLA-4 and TIM-3 (40, 45).

These findings suggest that the inhibition of CK2 can enhance antitumor immunity by downregulating the expression of PD-L1 on multiple tumor cells or DCs. Apart from PD-L1, the relationship between CK2 activity and other immune checkpoints needs further investigation.

## 5 Discussion

This review article comprehensively expounded the correlation between CK2 and tumor-immunity. The upregulation of CK2 can be found in many tumors and is closely related to the prognosis. By consulting relevant literature, we conclude that ①CK2 can regulate the growth and development of the immune cells in the tumor microenvironment (TME) both in innate and adaptive immunity.②CK2 also plays an essential role in regulating the production of cytokines to modulate the function of immune cells in the tumor microenvironment.③CK2 may also have a potential role in regulating oncogenic signaling pathways to affect these immune cells in TME. ④CK2 activity can regulate the expression of PD-L1 on tumor cells and DC cells, and the combination of CK2 inhibitors and immune checkpoint inhibitors can synergistically inhibit tumor growth. We also collated these conclusions (**Table 1**). Therefore, we can conclude that CK2 plays a vital role in tumor immunity at multiple sites, and targeting CK2 can significantly improve the anti-tumor immune effect.

**Table 1:** Protein kinase CK2 and tumor immunity.

**Tumor immunity**	**Signal pathway**	**Function**
**MDSCs and TAMs**	Notch signal	CK2 activity leads to defective DC cells differentiation and increased production of PMN-MDSC and tumor-associated macrophages (TAMs) in tumors (35, 40). CK2 can regulate the differentiation of the precursors of granulocytes and macrophages (40).
**DC cells**	-	CK2 can regulate the expression of PD-L1 on tumor-associated DCs and affect DC cells’ function to prime T cells (45).
**NK cells**	**-**	CK2 inhibitors can augment the cytotoxicity of NK cells (46, 47).
**CD4+ T cells, CD8+T cells, Treg cells**	Ikaros signal	CK2 activity increases Tregs and reduces the effector CD8^+^T and CD4^+^ T cells (48, 56).
**B cells**	BCR signal and Notch2 signal	CK2 in the development and differentiation of normal B cells (58).
**cytokines**	JAK/STAT3 and Notch signal The SHIP-1 expression	CK2 inhibitors can reduce the release of TNF, IL-6, and VEGF cytokine in cancer cells (73). CK2 inhibitors can suppress the production of tumor-derived factors (TDF) to modulate immune cells development and promote tumor regression (69).
**PD-L1**	**-**	CK2 activity can regulate the expression of PD-L1 on tumor cells (45).

Considering that CK2 has a huge regulatory effect on a variety of immune cells in multiple inflammatory diseases. Above all, the most striking function of CK2 is to modulate the Th17/Treg axis in CD4+ T cells in the model of autoimmune encephalomyelitis (24, 81). E.N. Benveniste et al. show that CK2 inhibitor CX-4945 treatment can suppress the maturation of Th17 cells, CK2α depletion impaires Th17 cell differentiation and promotes the production of Foxp3Tregs *in vitro (* 81). However, we know that Tregs are immunosuppressive cells and play a negative regulatory role in antitumor immunity. The above findings suggest that CK2 inhibitors cause the accumulation of Tregs in the inflammatory microenvironment, which would seem to be detrimental to anti-tumor immunity and is contrary to the CK2 inhibitors’ anti-tumor immune effect that we discussed. Therefore, whether CK2 inhibitors have a “double-edged sword effect” on the tumor immune microenvironment deserves further exploration.

In this review, we only introduce the effects of CK2 on the part of the immune cells in the TME in current research progress, while its effects on other cells and the specific regulatory mechanisms involved need to be further studied. In addition, the specific mechanism of how CK2 regulates the expression of immune checkpoints, such as PD-1/TIGIT/CTLA-4 in tumors, has not very clear. The biological function of CK2 is complex, and a deeper understanding of its function in immune and tumor cells will give more evidence to determine the effective CK2 inhibitors to treat solid and hematological tumors.

Nevertheless, this review still provides us with good evidence for the relationship between CK2 and tumor immunity. It offers more theoretical basis for targeting CK2 to improve the anti-tumor immune effect and promote the efficacy of immunotherapy to treat cancers.

## Author contributions

RM and ShZ conceived the study, LC and SiZ collected the literature, QL and JL drafted and revised the manuscript, and HD prepared the figures and participated in writing. All authors contributed to the article and approved the submitted version.

## Funding

Foundation for Fostering Key Talents from Middle-aged and Young Medical Personnel in Wuhan (2016), CSCO Cancer Research Fund (NO. Y-2019Genecast-061 and NO. Y-sy2018-018), 2019 Wu Jieping Medical Foundation- Xinda Cancer Research Fund.

## Conflict of interest

The authors declare that the research was conducted in the absence of any commercial or financial relationships that could be construed as a potential conflict of interest.

## Publisher’s note

All claims expressed in this article are solely those of the authors and do not necessarily represent those of their affiliated organizations, or those of the publisher, the editors and the reviewers. Any product that may be evaluated in this article, or claim that may be made by its manufacturer, is not guaranteed or endorsed by the publisher.
